# Comparıson of the Heart Rate and Blood Lactate Responses of Different Small Sided Games in Young Soccer Players †

**DOI:** 10.3390/sports4040048

**Published:** 2016-09-29

**Authors:** Yusuf Köklü, Utku Alemdaroğlu

**Affiliations:** Faculty of Sport Sciences, Pamukkale University, Denizli 20160, Turkey; utkualemdaroglu@yahoo.com

**Keywords:** intermittent exercise, game based training, RPE, internal responses

## Abstract

The purpose of this study was to compare the percentage of maximum heart rate (%HRmax), blood lactate (La^−^), and rating of perceived exertion (RPE, CR-10) responses across different formats of small-sided games (SSG) in elite young soccer players. Fourteen players (average age 16.7 ± 0.6 years; height 177.6 ± 4.1 cm; body mass 66.3 ± 4.7 kg; average training age 6.7 ± 1.6 years; percentage of body fat 8.4 ± 2.6%) volunteered to perform the YoYo intermittent recovery test (level 1) and eight bouts of soccer drills including 2-a-side, 3-a-side, and 4-a-side games without goalkeepers in random order at two-day intervals. Heart rates were monitored throughout the SSGs, whereas the RPE and venous blood lactate were determined at the end of the last bout of each SSG. The differences in La^−^, %HRmax, and RPE either across the different SSGs or between the bouts were identified using 3 × 8 (games × exercise bouts) two-way analysis of variance with repeated measures. Significant differences were found in terms of La^−^, RPE, and %HRmax among the different types of SSG (*p* ≤ 0.05). 3-a-side and 4-a-side games elicited significantly higher responses than 2-a-side games in terms of %HRmax (*p* ≤ 0.05), whereas 4-a-side games resulted in significantly lower La^−^ and RPE responses compared to 2-a-side and 3-a-side games. The results of this study show that physiological responses differ according to the numbers of players involved in small-sided games. Therefore, it can be concluded that 3-a-side and 4-a-side games could be more effective in improving high intensity aerobic performance than 2-a-side games, which in turn are more appropriate for developing anaerobic performance.

## 1. Introduction

As a game which requires jumping, shooting, challenges, turns, dribbles, sprints, controlling the ball under pressure, running at different speeds, and sliding tackles, effective performance in soccer is dependent upon both aerobic and anaerobic metabolism [1]. During a soccer match, players perform around 1000–1400 of these different actions and cover between 8.5 and 13.5 km [2,3,4]. This indicates that numerous factors might affect soccer performance and various player characteristics (technical, tactical, and physical) must therefore be developed in order to achieve top performance during matches. For this reason, it is important to be aware of the playing format that is best suited to developing the desired characteristics.

Small-sided games (SSGs) are one of the most popular types of match specific training. Their specific advantage and the reason why they have been increasingly used by coaches is that by using the same training drill, it is possible not only to develop technical capacities, such as players’ ability to keep the ball under pressure, but also to improve their aerobic endurance by recreating the type of physiological burden encountered during matches. However, when organizing SSGs, coaches who want to achieve and maintain the intensity of exercise that will best develop aerobic endurance may need to consider a number of different factors. These factors include the number of players and the pitch size [5,6,7], bout duration [8], game rules [9,10], coach encouragement [11], absence or presence of goalkeepers [12,13], team formation [14] and the training regime [15,16].

Coaches can use SSGs not only during the season for high intensity training, but also before for pre-season training by increasing the number of players. For example, Little [17] stated that small-sided games which involve more players such as 5-, 6-, 7-, and 8-a-side games can be used to develop the anaerobic threshold (85%–90% of HRmax). At the same time, 2-, 3-, and 4-a sided games are often used by coaches as high intensity aerobic training. Rampinini et al. [11] revealed that increased pitch size during three bouts of 3-a-side, 4-a-side, 5-a-side, and 6-a-side games resulted in increased heart rate and blood lactate responses in amateur soccer players. Meanwhile, Koklu et al. [5] demonstrated that decreasing the number of players results in increased intensity during small-sided games including six bouts of 2-a-side, 3-a-side, and 4-a-side games. Although Little [17] recommended to organize SSGs from 1 to 8 bouts, only the effects of different formats of SSGs from 1 to 6 bouts (continuous or intermittent) have been investigated by a number of studies [15,16]. On the other hand, intermittent SSGs are more suitable than continuous SSGs because of the passive rest period between each interval bout which may have allowed a greater physiological recovery, and intermittent SSGs elicited significantly more moderate and higher-speed running [15]. However, no studies were found that have investigated the effects on physiological response of SSGs of greater than 6 bouts. It is thought that more information about these effects will be useful to coaches in planning their training regimes, which provides the rationale for this study. This aim was addressed by comparing the percentage of maximum heart rate (%HRmax), blood lactate (La^−^), and rating of perceived exertion responses across eight bouts of SSGs in elite young soccer players.

## 2. Method

### 2.1. Subjects

Fourteen young soccer players (average age 16.7 ± 0.6 years; height 177.6 ± 4.1 cm; body mass 66.3 ± 4.7 kg; average training experience 6.7 ± 1.6 years; percentage of body fat 8.4 ± 2.6%; HRmax 195.7 ± 7.4 beat·min^−1^) voluntarily participated in this study. All the players were members of the same team competing in the top domestic league and they all trained for 90 min, five days per week. Written informed consent was obtained from all the subjects and their parents. The players and their parents were notified of the research procedures, requirements, benefits, and risks before giving informed consent. The study was approved by the Pamukkale University Ethics Committee, and was conducted in a manner consistent with the institutional ethical requirements for human experimentation in accordance with the Declaration of Helsinki.

### 2.2. Procedures

A 2-week training period was used to familiarize participants with the testing procedures and SSG formats. At the end of the familiarization period, players underwent the Yo-Yo intermittent recovery test level 1 (YIRT) and were ranked according to the distance covered in this test. The ranking system worked as follows: players who covered the least distance were given a score of 1 and those who covered the most distance were given a score of 5. The coach also provided a subjective rating of the overall technical/tactical skill level for each player using a 5-point scale (from 1 = poor to 5 = excellent). The total score for each player was the sum of their technical/tactical skill and YIRT scores [16,18,19]. In an attempt to avoid skill and fitness mismatches and a subsequent imbalance in opposing SSG teams, each SSG team was then balanced using the total score of the players [14].

This study was carried out over a 2-week pre-season training period in September 2012–2013, during which the fourteen young soccer players who participated were not involved in any other training or matches. On the first day (Tuesday), anthropometric measurements (height, body mass, skinfold thickness, circumference measurements) were taken and the YIRT level 1 test was carried out on the players. The HRmax for each player was determined during the YIRT. Starting on the third day, players performed different SSGs, either 2-, 3-, or 4-a-side games. Thereafter, each SSG session was separated by at least 2 days. In addition, each SSG was played after a 20-min standardized warmup session, which consisted of low intensity running, striding, and stretching. During the SSGs, HR responses were recorded. Rating of perceived exertion (RPE) surveys were presented to each player immediately after the end of the last bout of each SSG and La^−^ was determined 3 min after the end of the last bout of each SSG. The YIRT and SSGs were performed on a synthetic grass pitch at a similar time of the day (16:00–18:00) in order to have similar chronobiological characteristics [20]. To avoid any potential confounding effects of excessive wind or changes in temperature, YIRT tests and SSGs were only performed in clear and good weather conditions (temperatures from 20 °C to 25 °C, without rain or wind).

### 2.3. YoYo Intermittent Recovery Test

The YIRT consists of repeated 20-m runs back and forth between the starting, turning, and finishing lines at a progressively increasing speed, which is controlled by audio bleeps from a tape recorder. The test was performed on a synthetic grass field in groups of 6 players, as suggested by Bangsbo et al. [21]. Each player’s HR was measured and stored using Polar S810 HR monitors (Polar Electro OY, Kempele, Finland) throughout the test. Stored data were transferred to a computer and filtered by Polar Precision Performance SoftwareTM (PPP4, Kempele, Finland). The highest HR measurement was recorded as YoYo HRmax. 

### 2.4. Small-Sided Games

Table 1 shows the number of bouts, bout duration (min), pitch dimensions (length × width), and the rest between bouts for the SSGs. The SSGs were played on a synthetic grass soccer pitch with four supporting players situated out of the playing area. Teams were instructed to maintain collective possession of the ball for the longest time possible; no goalkeepers were involved. To ensure that the game would restart immediately if the ball left the field of play, spare balls were kept all around the perimeter of the pitch. The coaches continually offered verbal encouragement to the players during the games. Players were allowed to consume drinking water made available during the recovery periods between each SSG bout. Before the SSGs, players were informed about how many bouts would be played.

### 2.5. Heart Rate Monitoring

HRmax for each player was determined during the YIRT [22], and corresponded to the highest values of HR reached during the test. HR was recorded at 5-second intervals during SSG bouts using Polar S810 HR monitors (Polar Electro OY, Kempele, Finland). Exercise intensities during SSG bouts were assessed using HR, expressed as a percentage of the HRmax as measured in the YIRT test. The mean HR for the SSGs was calculated by taking the means of the eight bouts (recovery durations between bouts were removed). The HR data were expressed as a percentage of the HRmax.

### 2.6. Blood Sampling

Blood lactate samples (La^−^) were taken 3 min after the end of the last bout of each SSG in line with the recommendations of Taoutaou et al. [23]. The samples were taken from the ear lobes and were immediately analyzed using a Lactate Plus analyzer (Nova Biomedical, Waltham, MA, USA) which had been previously calibrated and validated [24]. Reliability for a Lactate Plus analyzer was strong (*r* = 0.99, *p* < 0.05) [25].

### 2.7. Rating of Perceived Exertion (RPE)

The CR-10 rating of the perceived exertion rating scale proposed by Foster et al. [26] was presented to each player immediately after the last bout of each SSG. All players were informed about, and familiarized with, the CR-10 scale before the SSGs. This scale has been previously validated as an indicator of training intensity in intermittent tasks of SSGs [27].

### 2.8. Statistical Analysis

All data are reported as means and standard deviations. Before using parametric tests, the assumption of normality was verified using the Shapiro-Wilk test. The differences in La^−^, %HRmax, and RPE between either the small-sided games or the bouts were identified using a 3 × 8 (games × exercise bouts) two-way analysis of variance with repeated measures. The level of statistical significance was set at *p* < 0.05. Effect sizes (η^2^) were also calculated and values of 0.01, 0.05, and above 0.15 were considered small, medium, and large, respectively [28]. In addition, inter-individual variability of %HRmax, La^−^, and RPE responses across the 2-, 3-, and 4-a-side games were quantified using the coefficient of variation (CV). A 95% Confidence Interval (95% CI) around the mean was calculated for each variable.

## 3. Results

The average La^−^, %HRmax, and RPE responses of the soccer players in the eight bouts of each SSG are shown in Table 2. The lowest %HRmax responses were found in 2-a-side games, while the 3-a-side games resulted in the highest %HRmax responses. The lowest La^−^ and RPE responses were found for the 4-a-side games, whereas the highest responses were found for the 2-a-side games. One-way repeated ANOVA showed statistically significant differences between 2-, 3-, and 4-a-side games in terms of %HRmax (F = 17.807, *p* = 0.001, η^2^ = 0.587; large effect), La^−^ responses (F = 18.034, *p* = 0.001; η^2^ = 0.581; large effect), and RPE responses (F = 23.146, *p* = 0.001; η^2^ = 0.640; large effect). 

**Figure 1:**
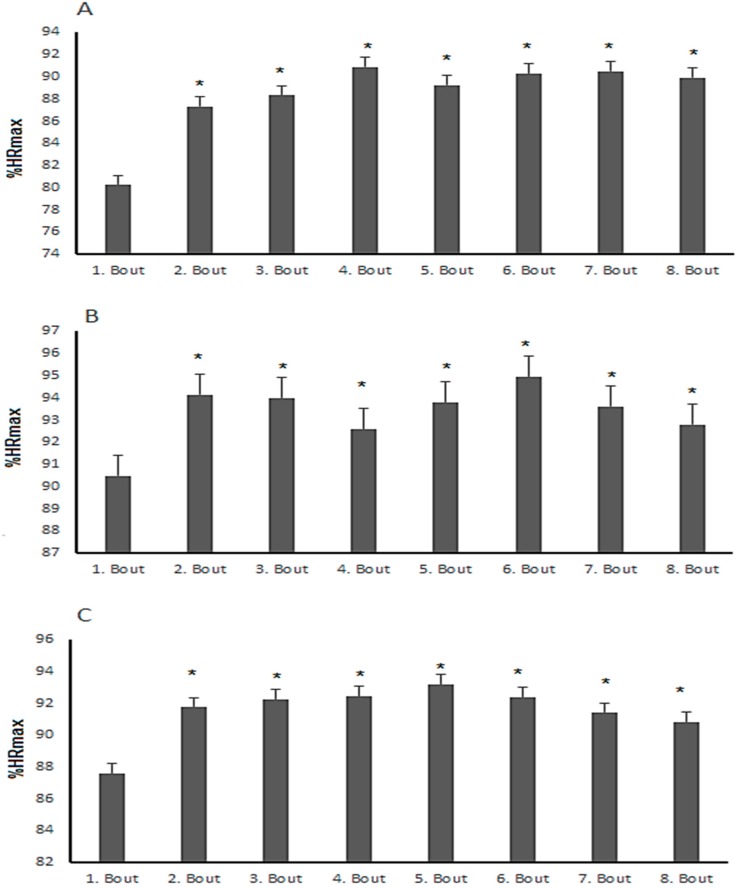
%HRmax responses of the soccer players for the eight bouts played in the (**A**) 2-a-side; (**B**) 3-a-side; and (**C**) 4-a-side games; * Significant difference from the first bout, *p* < 0.05.

Post-hoc pairwise comparisons revealed that significant differences were found in La^−^, HR, and %HRmax between SSGs (*p* ≤ 0.05). In terms of %HRmax, 3-a-side and 4-a-side games elicited significantly higher responses than 2-a-side games (*p* ≤ 0.05). In contrast, 4-a-side games resulted in significantly lower La^−^ and RPE responses compared to 2-a-side and 3-a-side games. In addition, the %HRmax response during the first bout was significantly lower than the other seven bouts in all formats, as shown in Figure 1.

## 4. Discussion

The aim of this study was to compare the percentage of maximum heart rate (%HRmax), blood lactate (La^−^), and RPE 10 responses between different SSG formats in elite young soccer players. The main findings show that physiological responses differ across the formats after eight bouts of each format.

The mean %HRmax value for the SSGs ranged between 88.3 and 93.3 %HRmax in the current study. These values are similar to those in previous studies [15,16,29,30,31,32]. This suggests that eight bouts of 2-a-side, 3-a-side, and 4-a-side games could be used for the improvement of soccer specific aerobic endurance in young soccer players. The present study also demonstrated that %HRmax during the first bout was lower compared to the other seven bouts in all formats. In addition, in all formats, there were low CVs in %HRmax responses across the format (ranging from 2.6% to 3.8%). These results demonstrate that players’ %HRmax responses were homogeneous within each format, perhaps partly because the ability of each team was balanced by the total score of fitness and technical/tactical skills in this study. These results are similar to those in previous studies [11,33]. In addition, the study results revealed that eight bouts of 3-a-side and 4-a-side games resulted in significantly higher %HRmax responses than eight bouts of 2-a-side games (*p* ≤ 0.05). This result could be because these game formats involve more players over a longer playing time and therefore less time on the ball compared to 2-a-side games. Additionally, the lactic acid system is more dominant in 2 a-side games than the other formats that can also cause less HR% responses. This may also suggest the reason why 3-a-side and 4-a-side games result in higher aerobic response.

Although HR monitoring is a frequently used method to monitor exercise intensity, it has some limitations. For example, while HR may overestimate the energetic cost of exercise, it may also underestimate the intensity of the very short bouts of intermittent exercise which characterize SSGs [34]. Therefore, exercise intensity in SSGs is not only established by measuring players’ heart rate (HR) responses during the game, but also by utilizing post-SSG RPE and blood lactate responses [27]. In the current study, mean La^−^ responses of SSGs ranged between 8.2 mmol·L^−1^ and 11.0 mmol·L^−1^ and RPE responses ranged between 6.3 and 8.1. These values are similar to those reported in previous studies [11,16,30]. The current study also revealed that large CVs were observed in RPE (ranging from 11.4% to 14.3%) and La^−^ (ranging from 19.6% to 26.4%) responses when the same SSG format was played. These results demonstrate that large differences exist between players with respect to RPE and La^−^ responses during SSGs. These findings also reveal inter-player variability relating to using different formats as a training stimulus in terms of RPE and La^−^ responses, a finding also reported in previous studies [11,13]. In addition, 2-a-side games resulted in significantly higher La^−^ and RPE responses compared to 3-a-side and 4-a-side games. The reason for this result may be that having fewer players and a smaller playing area means that players in 2-a-side games are involved in more one-on-one challenges and more changes of speed.

The most important limitation of this study is that measures of technical actions such as tackles, winning the ball from an opponent, or pass completion rates were not included. Another limitation is that we were not able to measure the distance covered at various running speeds. In addition, pacing strategies during intermittent activities could be influenced by different factors [35,36] and the number of bouts could be one of these factors; unfortunately, we did not consider pacing strategies in this study.

## 5. Conclusions

The results of this study showed that physiological responses over eight bouts of SSGs differed according to the format of the game. Higher heart rate and lower blood lactate concentration were found in 3-a-side and 4-a-side games compared to 2-a-side games. This indicates particularly that 2-a-side games have higher lactic anaerobic characteristics, whereas 3-a-side and 4-a-side games have greater aerobic characteristics. Therefore, it can be concluded that 3-a-side and 4-a-side games are more effective in improving high intensity aerobic performance than 2-a-side games, which seem more appropriate for developing anaerobic performance.

Also coaches should take in account of number of bouts in SSGs. This study showed that coaches could use the number of bouts as a training tool. Coaches could organize training with more players and less bouts numbers in the second part of the preparatory period, and then they could increase the intensity of training by either decreasing the players’ number or increasing the number of bouts.

## Figures and Tables

**Table 1:** Characteristics of small-sided games used in this study.

Drill	Duration (min)	Number of Bouts	Rest between Bouts (min)	Pitch Size (W × L)
2-a-side	2	8	2	12 × 24 m
3-a-side	3	8	2	18 × 30 m
4-a-side	4	8	2	24 × 36 m

**Table 2:** Average La^−^, HR, and %HRmax responses for the different small-sided game formats.

Variables	2-a-Side Games			3-a-Side Games			4-a-Side Games		
	Mean ± SD	CV (%)	95% CI	Mean ± SD	CV (%)	95% CI	Mean ± SD	CV (%)	95% CI
%HRmax	88.3 ± 3.3 *^,Ω^	3.8	86.4 to 90.3	93.3 ± 2.4	2.6	91.9 to 94.7	91.5 ± 2.4	2.7	90.1 to 92.9
La^−^ (mmol·L^−1^)	11.0 ± 2.9 *^,Ω^	26.4	9.3 to 12.7	9.1 ± 1.8	19.6	8.1 to 10.1	8.3 ± 1.7	20.5	7.3 to 9.2
RPE (CR-10)	8.1 ± 1.2	14.3	7.5 to 8.8	7.4 ± 0.8	11.4	6.9 to 7.8	6.3 ± 0.7 ^¥,^*	11.6	5.9 to 6.7

%HRmax: Percentage of maximum heart rate; La^−^: Blood lactate; RPE: Rating of perceived exertion; ^¥^ Significant difference from 2-a-side games, *p* < 0.05; * Significant difference from 3-a-side games, *p* < 0.05; ^Ω^ Significant difference from 4-a-side games, *p* < 0.05.
